# Perceptions about Authentic Leadership Development: South African Occupational Therapy Students' Camp Experience

**DOI:** 10.1155/2018/1587906

**Published:** 2018-03-25

**Authors:** Fatima Hendricks, Susan Toth-Cohen

**Affiliations:** ^1^OTASA, Vlaeberg, P.O. Box 15809, Cape Town 8018, South Africa; ^2^Department of Occupational Therapy, Thomas Jefferson University, 130 S. 9th Street, 632 Edison, Philadelphia, PA 19107, USA

## Abstract

**Background:**

Twenty-three years into democracy, concern is deepening regarding the slow progress of Occupational Therapy (OT) in South Africa, especially with regard to diversity and inclusion within OT.

**Methods:**

This study explores authentic leadership development primarily among Black OT students attending a pilot Occupational Therapy Association of South Africa (OTASA) National Student Leadership Camp. It seeks to ascertain their perceptions on leadership and leadership development. This descriptive pilot study employs in-depth interviews and subsequent content analysis, with 12 OT students from six university OT programs in South Africa.

**Findings:**

Four categories of participant perceptions on authentic leadership development emerged from the analysis: (1) perceptions about oneself as a leader based on personal narrative, self-awareness, self-control, and psychological capital; (2) perceptions about others, specifically current leaders, with regard to their moral crisis, including continuing inequality, insincerity, greed, and selfishness; (3) goals and aspirations for leadership development via student camps; and (4) effects of leadership on the system.

**Conclusions:**

Recommendations for future practice include promotion of storytelling as a means of personal reflection for authentic leadership development and focused investment in camps for developing student leadership skills and building authentic leadership knowledge.

## 1. Introduction

The South African OT community continues to grapple with issues related to the slow pace of social transformation, particularly racial transformation, diversity, and inclusion [1]. Exploring emerging OT student leaders' perceptions of their leadership journeys and engagement with authentic leadership may provide insight into student leaders' implicit theories of leadership in a postcolonial context. This, in turn, may enable greater understanding of OT students' leadership development needs and lead to a more informed approach to developing transformational plans to enhance diversity within the OT profession in South Africa.

As per OTASA's vision for 2020, students are an important strategic driver in the OT community in South Africa for their potential contribution to societal transformation [2], and, as OTASA's future leadership pipeline at the branch and national level, they reflect diversity and inclusion.  Table 1 represents the number of OT students registered with the Health Professions Council and their demographic profiles [3]. Approximately 12% of the students are members of OTASA, and there is significant opportunity for growth in student membership and participation [1].

The data in Table 1 reveal that the racial profile of the students relative to South Africa's demographics is skewed toward minority group overrepresentation, with 997 White students in OT programs compared with 773 Black African, 283 Colored, 167 Indian, and 2 Chinese students. At the same time, the number of Black students enrolling in OT programs is increasing, also illustrated in Table 1 [3]. Black African OT student admission has increased by 26.7% from 2013 to 2016 when compared to a 6.4% increase in White students during the same period. Diversity and inclusion to promote an Occupational Therapy community that reflects the demographics of the country is a key issue in the preliminary results of OTASA's National Listening & Dialogue Campaign, a series of ongoing national consultative workshops [4]. From these discussions, it is evident that gaps exist in engaging emerging Black OT leaders among students, hence the strategic importance of the National Student Leadership Camp and its focus on authentic leadership development for Black emerging student leaders.

Another strategic drivers for transformation, diversity, and inclusion is the recent student protests, manifested by #feesmustfall and #rhodesmustfall, which have been accompanied by a vibrant student movement reemphasizing the role of students in leading change for accessible and relevant education [5]. In addition, there is the upcoming World Federation of OT (WFOT) Congress 2018, to be hosted in South Africa, titled “Connected in Diversity: Positioned for Impact” [6], which requires student engagement across the racial and social spectrum as a prerequisite for its success.

Hence, to engage emerging Black student leaders in leadership development, a pilot leadership camp was conducted to meet the strategic needs of the OT community and promote diversity within the profession. The residential student leadership camp drew primarily emerging Black student leaders from OT programs across the country. The research question for this pilot study focused on examining individual student perceptions of authentic leadership development during camp participation; hence, personal narrative in-depth interviews were conducted with OT student camp participants.

Authentic leadership [7–12] was chosen as the organizing framework for the pilot study, because it includes a developmental focus appropriate for emerging leadership in students [13]. At its core, authentic leadership contains key concepts related to the self—such as self- awareness, self-knowledge, self-regulation, and personal values—which can be controlled by students desiring to effect change. Walumba et al. [9] describe four dimensions of authentic leadership: self-awareness, balanced processing, relational transparency, and internalized moral perspective; these are significant in the context of constructing a life story narrative, as elucidated by Shamir and Eilam [8]. Self-awareness is the extent to which leaders know and understand their own true selves. Balanced processing refers to unbiased analysis of all relevant information and taking into account others' opinions and input before making a decision. Relational transparency involves openly sharing information and expressing one's true thoughts and emotions with others. Internalized moral perspective refers to the extent to which leaders' behaviors are guided by internal moral standards and values rather than by external pressures [14].

Shamir and Eilam [8] also define four characteristics of authentic leaders: they are (1) true to themselves (rather than conforming to the expectations of others); (2) motivated by personal convictions, rather than by status, honor, or other personal benefits; (3) original, leading from their own personal point of view; and (4) spurred to action based on their personal values and convictions.

The construction of a life story is a major element in the development of authentic leadership. Shamir and Eilam [8] further argue that authentic leadership “rests heavily on the self-relevant meanings the leader attaches to his or her life experiences, and these meanings are captured in the leader's life-story.” They suggest that self-knowledge, self-concept clarity, and person-role merger are derived from the life story, making the construction of life story critical to the development of authentic leaders. This model provides a meaningful system of feeling, thinking, and acting [8], which is particularly relevant in the evolving sociopolitical, historical, and cultural contexts of South Africa. Hence, this pilot study aims to fill an important gap by focusing on authentic leadership development for primarily emerging Black OT student leaders by asking them how they perceive authentic leadership and its development. The descriptive findings from the study may contribute to theory development by revealing information about Black South African OT students' implicit theories of leadership. Greater understanding of these implicit theories of leadership may then inform development of strategies to help Black OT students in South Africa to further develop their leadership capacity and contribute their unique perspectives to leadership within the OT profession.

## 2. Materials and Methods

### 2.1. Aim of the Study

This descriptive pilot study explored the personal narratives of Black OT students attending the first OTASA National Student Leadership Camp designed to promote authentic leadership development, from September 23 to 25, in Limpopo, South Africa.

## 3. Method

The project was approved by the Institutional Review Board (IRB) of the authors' university and was part of a larger study using mixed methods to explore leadership development among South African OT students. Data were gathered from structured interviews based on key concepts of authentic leadership [7, 8, 10–12, 15].

Participants comprised 12 Occupational Therapy students from six university OT programs in South Africa. The purposive sample was drawn from first- to third-year OT students (*n* = 34), who attended the first pilot National Student Leadership Camp prior to commencement of the camp (see demographics in Table 2). Two students from each university were selected for the interviews, and six of the eight OT programs in South Africa were represented.

### 3.1. Data Collection and Analysis

*Data Collection. *A structured interview questionnaire was used, consisting of six questions based on the principles of authentic leadership according to Gardner et al. [11] and Shamir and Eilam [8]: life story and life purpose, positive psychological capital, positive moral perspective, self-regulation, self-awareness, and aspirations for the student leadership camp; these aspects were developed as a discussion guide. The questionnaire was developed from the principles of authentic leadership and formulated into a grid of key elements of authentic leadership, like positive psychological capital and other elements, to serve as a rubric. The rubric assessment for categorizing their responses as awareness, exploration/engagement, or generativity was then used after each response was recorded (see Table 3). Responses were captured in writing by the first author prior to student participation in the camp.

*Data Analysis. *Data analysis began with open coding of participants' survey responses using NVivo. Constant comparative analysis within and across survey data allowed the researchers to reduce categories and create a structured coding system through organizing and coding with nodes in trees in NVivo [16]. The investigators completed the coding processes separately and compared coded statements that demonstrated strong consensus, hence establishing intercoder reliability.

## 4. Results and Discussion

**Figure 1:**
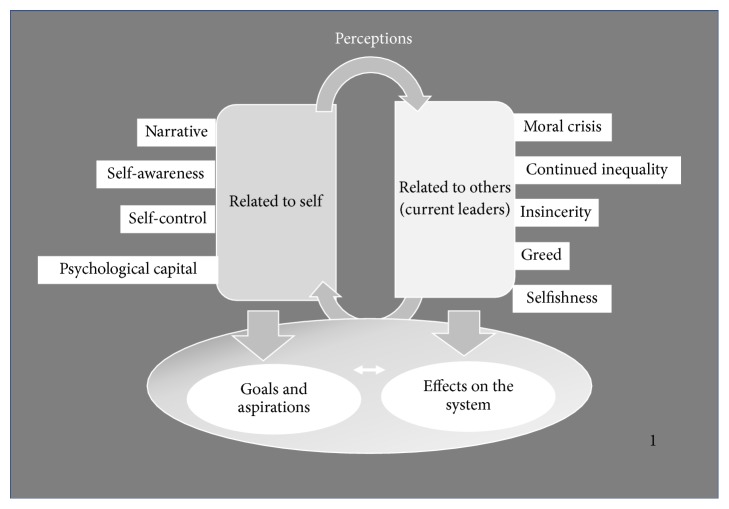
Student perceptions of authentic leadership.

The findings of this study revealed participants' perceptions of leadership as reflective of their internalized beliefs and expectations of leaders (implicit leadership theories). The data in Figure 1 show that the primary themes can be divided into four main categories: (1) perceptions of authentic leadership in relation to self; (2) perceptions of key issues with current leaders; (3) goals and aspirations for authentic leadership development via camps; and (4) effects of leadership on the system.

### 4.1. Grading Rubric

The rubric outlined in Table 3 was based on the core elements of authentic leadership [8, 11]. This rubric was used to grade each participant according to the quality of their responses in terms of the awareness, exploration, or generativity levels for each of the five key questions asked, the sixth being a question on camp goals and aspirations.

For Question 1, 67% of the participants were found to be on the exploration level because they were able to briefly describe leadership components in relation to life goals or life story, whereas 33% were graded at the generativity level for their ability to engage in a thorough discussion of life goals or life story relating to the key components of leadership. In terms of the framework, authentic leaders are self-concordant individuals who pursue life goals expressing their authentic choices rather than following externally imposed duties or conventions and are motivated by internal commitment, which is a commitment to a self-concept [8].

Regarding Question 2, 83% were graded at the exploration level for demonstrating some understanding of the psychological competencies for becoming an authentic leader, whereas 17% were placed at the generativity level for demonstrating understanding that having confidence, a vision of success, perseverance, and resilience are key psychological competencies for becoming an authentic leader. According to Luthans et al. [17], an individual's positive psychological state of development is characterized by (1) confidence, (2) being optimistic about succeeding, (3) perseverance toward goals and, when necessary, redirecting paths toward goals (i.e., showing hope) in order to succeed, and (4) when beset by problems and adversity, sustaining and bouncing back and even beyond (i.e., resilience) to attain success.

For Question 3, 8% of participants were graded at the awareness level for their ability to suggest some core ethical issues in relation to leadership and moral capacity, moral courage, and resiliency; 58% at the exploration level for discussing, in some detail, core ethical issues in relation to leadership and moral capacity, moral courage, and resiliency; and 33% at generativity for their ability to analyze, with depth and clarity, core ethical issues in relation to leadership and moral capacity, moral courage, or resiliency. In terms of positive moral perspective, May et al. [18] provide an extensive discussion of this moral component, describing an ethical and transparent decision-making process whereby authentic leaders develop and draw upon reserves of moral capacity, efficacy, courage, and resiliency to address ethical issues and achieve authentic and sustained moral actions.

Among the participants for Question 4, 8% were graded at the awareness level for referring to certain details on the importance of self-control and the need to assess and manage alternate, divergent, or contradictory perspectives or ideas in comparison with internal standards. At the exploration level, 58% were graded for being able to highlight the importance of self-control and the need to assess and manage alternate, divergent, or contradictory perspectives or ideas in comparison with internal standards in an exploratory way, whereas 33% were graded at the generativity level. Self-regulation involves processes whereby people exert self-control by (1) setting internal (either existing or newly formulated) standards, (2) assessing discrepancies between these standards and actual or expected outcomes, and (3) identifying intended actions for reconciling these discrepancies [19].

George [20] describes self-awareness as an emerging process and not a destination point, in which individuals continually come to understand their unique talents, strengths, sense of purpose, core values, beliefs, and desires that include basic and fundamental awareness of their knowledge, experience, and capabilities. Gardner et al. [21] further describe four components: values, cognitions regarding identity, emotions, and motives/goals. Thus, among the participants for Question 5, 8% were graded at the awareness level for mentioning some strengths and weaknesses but with little connection to life goals or purpose, values, identity, and emotions, whereas 67% of participants were able to analyze personal strengths and weaknesses with some connection to life goals or purpose, values, identity, and emotions (exploration level), and 25% of the participants were able to effectively analyze strengths and weaknesses with a strong connection to life goals or purpose, values, identity, and emotions (generativity level).

Overall, for each of the questions, over 90% of the participants were graded at the exploration or generativity levels. The next section outlines the content of the in-depth interviews in terms of key themes that emerged.

### 4.2. Key Themes

Figure 1 illustrates the four categories of participant perceptions that emerged on authentic leadership development: (1) perceptions about self as a leader based on personal narrative, self-awareness, self-control, and psychological capital; (2) perceptions about others, specifically current leaders, relating to their moral crisis, continued inequality, insincerity, greed, and selfishness; (3) goals and aspirations for leadership development via camps; and (4) effect of leadership on the system.

Figure 1 outlines the subthemes in relation to perceptions of authentic leadership pertaining to leadership of the self as follows: (a) life narrative—the self as leader/role model, the influence of family and significant others on leadership development, and the relationship among leadership, OT, and life purpose; (b) self-awareness—being true to self, conscious of self, and accepting of self; (c) self-control—self-mastery, leading carefully through careful thinking, emotional control, and using a moral compass; and finally (d) positive psychological capital—perseverance/resilience, positivity, and openness. For example, relating to perceptions of authentic leadership pertaining to self in the subtheme of positive psychological capital, one student said,

I have a lot of zeal and persistence to press forward. I could have given up but I never gave up. I have to be positive, and I have to make a contribution. (Black, female)

This statement is typical of the perceptions of the participants' highlighting their perseverance and resilience within the context of their personal life narratives.

Figure 1 also outlines the subthemes of critiquing current leaders in relation to their authentic leadership as follows: (a) moral crisis in leadership; (b) continued societal inequality as a serious leadership challenge; (c) insincerity of leadership; (d) greed and corruption; and (e) selfishness in looking out for one's own self-interests over the needs of the people. In a critique of current leaders on the subtheme of moral crisis, a student stated,

It is disconcerting with fraud, corruption and with misplaced values. I think it is because it is lonely at the top and they have become addicted to power and lost their moral compass. (Black, male)

Finally, Figure 1 outlines camp goals and aspirations for authentic leadership development via camps, the subthemes being listed as follows: (a) pursuit of leadership knowledge and skills, (b) aspirations for becoming a leader, and (c) building confidence for leadership. Students perceived themselves as active agents of or catalysts for change in the OT profession because, by pursing their leadership goals and aspirations, they could effect positive change in the societal system. As an example of a statement representing camp goals and aspirations for authentic development as a leader through leadership camps, a student noted,

When I work with children, I look at them and say to myself—look at that dance, it's so imperfectly perfect. And that's what I want to be to the children—to sincerely help them, encourage them. It starts with the self—this commitment and desire I have to do good. We forget about ourselves in terms of pretending to care but rather the leadership must come from within. The way I lead will be different after this camp. I will spend time reflecting and evaluating myself, and then I want to go back and share my camp experience with the rest of the class. (Colored, female)

In summary, the results indicate that over 90% of the participants were graded by the rubric at the exploration or generativity level in terms of exploring their authentic leadership development, and four themes emerged in terms of (a) perceptions of self as a leader, (b) perceptions of others, specifically current leaders, (c) goals and aspirations for leadership development using camps, and (d) effect of leadership on the system.

## 5. Discussion

**Figure 2:**
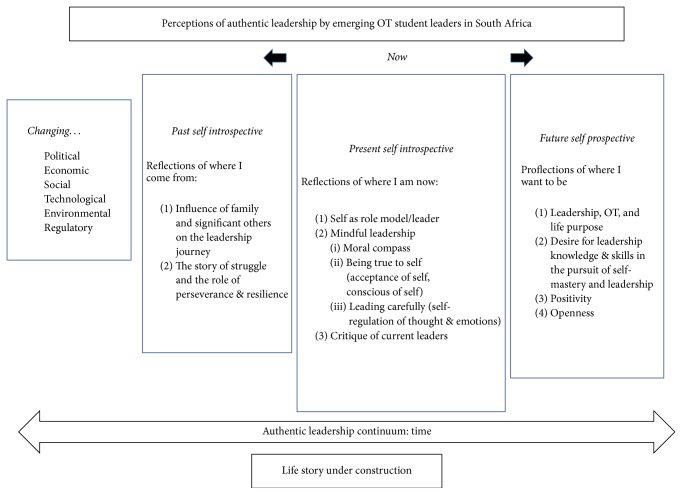
Working model of authentic leadership development for South African students.

A working model of the authentic leadership development model for students (Figure 2) is proposed based on the insights garnered from the results.

Preliminary findings from this pilot study indicate that the students are on a leadership continuum, given that they describe themselves retrospectively in terms of their “past self,” such as where they come from; in subthemes like the important role of family and significant others in leadership development; the story of their struggle; and the role of perseverance and resilience in shaping their journeys. Students spent a significant amount of time describing themselves in terms of their “present self,” such as in their reflections on role modeling, mindful leadership, and current leaders. The students “proflected”; that is, they began to consider the thoughts, feelings, and perceptions of their future, linking them to OT, life purpose, leadership, and a desire for leadership and self-mastery knowledge and skills, all the while valuing positivity and openness on the journey.

Interestingly, Erikson's developmental theory [22, 23] is not without its critics, especially in the area of identity formation and gender. Hoare [24] and Archer [25] describe the ages between 13 and 19, extending into the twenties, as the stage of fidelity in terms of identity versus role confusion in answering the questions, “Who am I, and what can I become?” It is characterized by an emerging sense of self through forging past experiences with anticipations of the future state with sufficient time for gaining clarity about values and convictions, exploring the role of leadership central to self-concept, developing self-resolution, and displaying self-expressive behavior with goals that are self-concordant [8].

Through a critical lens, this is where Shamir and Eilam's [8] proposition of authentic leadership development falters in presenting the notion of the life story as a “narrative” of leadership development. It is perhaps better considered as the life story “question” or “construction” rather than a “narrative” because, through a postcolonial lens, issues of contested identities exist [26], which have the potential to be in conflict with the concepts presented by both Shamir and Eilam [8] and Walumbwa et al. [14]: self-concept clarity, self-awareness, and self-knowledge for authentic leadership development. These concepts appear to be singular rather than hybrid or plural in nature. Postcolonial theories [26] present the notion that identities are multiple and evolving, whereas a self-concept is a combination of social categories comprising a set of social identities that is part of a collective of interwoven stories rather than an individual story—which in the South African context is called “Ubuntu.” Ubuntu became prominent in mainstream discourse on race and identity during the transition from Apartheid into democracy and raises two important principles: the human being as being becoming (not static); and the importance of community. Whether Ubuntu can be claimed as the way South Africans experience their lived social realities and its relationships, order, and identities is debatable; however it can be described as a discursive South African social imaginary. Taylor [27] defines social imaginary as “common understanding that makes possible common practices and a widely shared sense of legitimacy.” Ubuntu is important for the discussion of authentic leadership development in the context of the personal narrative of students' retrospective views of self in the past, introspective views of self in the present, and prospective views into the future shaping identities which are in a fluid state of construction and negotiation. This is consistent with the notion of authenticity of identity as contested at the intersection of racial and professional identities for OT students that was revealed in the narrative interviews.

Ubuntu is an African concept of philosophy according to which no one is self-sufficient, and all are interdependent:* umntu ngumntu ngabantu *means a person is a person because of others. Radically, Bujo [28] argues that an individual cannot be spoken about outside the notion of a community because “the human person in Africa is from the very beginning in a network of relationships that constitute his inalienable dignity,” and that “African ethics does not define the person as self-realization or as ontological act; rather, it describes a person as a process of coming into existence in the reciprocal relatedness of individual and community, where the latter includes not only the deceased but also God” [28].

Hence, the proposed model of authentic leadership development includes students'* retrospective *views of self in the past,* introspective *views of self in the present, and* prospective *views into the future. These views of leadership are considered aspects of students' implicit theories of leadership and located within the macro context of the ever-changing social web of political, economic, social, technological, environmental, and regulatory environments in South Africa.

Within the historical context of a contested description of being “Born Frees” (i.e., born into Democratic South Africa), students face significant challenges from the macro environment in terms of socioeconomic-political struggles, requiring positive psychological capital. Furthermore, the need for positive psychological capital is amplified by the myriad of ethical and moral crises facing leaders today. Students described these challenges as having leaders who are corrupt and self-serving, power hungry, and morally decayed—leaders who lack discipline and respect for the rule of the law and are greedy.

In summary, the potential benefits of the proposed model of authentic leadership development are as follows: (1) leadership development is ongoing, a continuum, from youth till late adulthood; (2) leadership development is located in particular times and historical contexts that shape leadership, its formation, and expression and is therefore dynamic and responsive; (3) personal storytelling in camps is valuable in deconstructing and reconstructing life stories, individually or collectively, to facilitate leadership growth in self-knowledge, self-awareness, and other elements of mindful leadership; (4) leadership development requires follower development in a mutually symbiotic way, in consideration of the collective; (5) leadership development recognizes life purpose, values, morality, and God as elements of authenticity; (6) leadership development employs a scoring rubric designed for the youth.

The insights build a bridge between the conceptual understanding of authentic leadership and student perceptions of Occupational Therapy student leadership development through camping as a means of OT student leadership capacity building. It advocates for retrospective, introspective, and prospective views of leadership on a personal level as an enabler for Occupational Therapy students to engage in their own personal and professional leadership development through a diversity and inclusion approach of targeted student leadership development.

### 5.1. Limitations

The qualitative interviews were limited to 12 students from six of the eight universities of South Africa, of whom seven were Black African participants. Participation from all eight OT universities while maintaining the same racial demographics is a consideration for future work.

## 6. Conclusions

This exploratory pilot study provides insights into the potential for authentic leadership development for OT students based on four categories of authentic leadership: (1) perceptions about self as a leader based on personal narrative, self-awareness, self-control, and psychological capital; (2) perceptions about others, specifically current leaders, relating to moral crises, continued inequality, insincerity, greed, and selfishness; (3) goals and aspirations for leadership development via camps, and (4) the effect of leadership on the system. Although the sample size was relatively small, these findings provide insights into the potential for authentic leadership development and can be a springboard for further elaboration of student leadership development, particularly for emerging Black student leaders in OT. Findings may also contribute to further theory development in regard to students' own implicit theories of leadership.

Recommendations for future research and practice associated with authentic student leadership development include (1) the important role of storytelling in shaping leadership development narratives after 1994 for the so-called “Born Frees” as a means of sense-making and dreaming—in search of answers to the questions, “From where? To where?”; (2) the need for specific focused investment in the development of Black OT student leadership and the harnessing of all tools available in the context of the roles that family, educators, and the environment play in shaping leadership development; (3) authentic leadership and authentic leadership development from an African perspective, with particular consideration for postcolonial theorizing; and (4) enhancing camp experiences as a means of youth leadership development in the health professions sector, particularly OT, based on activity participation and experiential learning.

In conclusion, the proposed new model for authentic leadership development for OT students in South Africa has been built on OT students' perceptions of key issues with current leaders, perceptions about authentic leadership in relation to self, and student goals and aspirations for authentic leadership development via camps. This model can help the OT profession gain more understanding of the leadership development needs of emerging young Black leaders and may assist in theory development related to implicit theories of leadership. For example, as part of a mixed methods study, a survey could be designed to gather additional data on South African OT students' attitudes and perceptions of Occupational Therapy student leadership—with particular focus on diversity and inclusion. Together with a continued focus on storytelling and qualitative analysis of student narratives, results of the survey could provide insights for the formulation of transformational and leadership skills development plans for the OT profession in South Africa.

## Figures and Tables

**Table 1:** Demographic profile of OT students (OT S) and membership in OTASA.

REG_CODE	Race	Dec	Dec	Dec	Apr
		2013	2014	2015	2016
OT S	African	610	673	678	773
	Chinese	1	1	2	2
	Colored	243	262	259	283
	Indian	136	148	152	167
	White	918	908	902	977
	(None)	80	87	88	99

OT S total		1,988	2,079	2,081	2,301

OTASA student membership		130	249	266	289

OTASA student membership%		6.54%	11.98%	12.78%	12.56%

*Source*. Health Professions Council of South Africa (2016); OTASA (2016).

**Table 2:** Participant demographics of personal narrative interviews.

Attribute	Description	*N* = 12
Gender	Male	5
	Female	7

Age	18–20	7
	21–23	3
	24–26	2
	27+	0

Race	Black	7
	Colored	2
	Indian	2
	White	1

Year of study	First	5
	Second	5
	Third	2
	Fourth	0

University	University of Cape Town	0
	University of Free State	0
	University of Kwa-Zulu Natal	2
	University of Pretoria	2
	Sefako Makgatho Health Sciences University	2
	University of Stellenbosch	2
	University of Western Cape	2
	University of Witwatersrand	2

**Table 3:** Grading rubric of the level of engagement with authentic leadership.

Question	Awareness	Exploration	Generativity	Percentage
(1) Life story and its relationship to leadership camp attendance	*N* = 0	*N* = 8	*N* = 4	Awareness: 0% Exploration: 66.66% Generativity: 33.33% 100% at exploration level or higher

(2) Psychological competencies for authentic leadership	*N* = 0	*N* = 10	*N* = 2	Awareness: 0% Exploration: 83.33% Generativity: 16.66 100% at exploration level or higher

(3) Ethical issues facing leadership today	*N* = 1	*N* = 7	*N* = 4	Awareness: 8.33% Exploration: 58.33% Generativity: 33.33% 91.66% at exploration level or higher

(4) Self-regulation and authentic leadership	*N* = 1	*N* = 7	*N* = 4	Awareness: 8.33% Exploration: 58.33% Generativity: 33.33% 91.66% at exploration or higher

(5) Self-awareness and personal examples	*N* = 1	*N* = 8	*N* = 3	Awareness: 8.33% Exploration: 66.66% Generativity: 25% 91.66% at exploration level or higher

## References

[1] OTASA Membership Office Membership demographics. Internal database.

[2] OTASA About, mission, vision & values. http://www.otasa.org.za/.

[3] Health Professions Council of South Africa (HPCSA) Student register.

[4] OTASA Western Cape National Listening & Dialogue Campaign Preliminary results: internal report.

[5] Badat S. Deciphering the meanings, and explaining the South African higher education student protests of 2015-16. https://www.ru.ac.za/media/rhodesuniversity/content/uhuru/documents/Dr%20Saleem%20Badat%20-%20The%20Student%20Protests%20of%202015-16%20Final%20Draft-10March2016.pdf.

[6] World Federation of Occupational Therapy WFOT congress. http://www.wfotcongress.org.

[7] Avolio B. J., Luthans F., Walumbwa F. O. (2004). *Authentic Leadership: Theory Building for Veritable Sustained Performance*.

[8] Shamir B., Eilam G. (2005). What’s your story? A life- stories approach to authentic leadership development. *The Leadership Quarterly*.

[9] Walumba F., Avolio B., Gardner W., Wernsing T., Peterson S. (2008). *Authentic Leadership: Development and Validation of a Theory-Based Measure*.

[10] Avolio B. J., Gardner W. L., Walumbwa F. O., Luthans F., May D. R. (2004). Unlocking the mask: a look at the process by which authentic leaders impact follower attitudes and behaviors. *The Leadership Quarterly*.

[11] Gardner W. L., Avolio B. J., Avolio B. J., Walumbwa F. O., Gardner W. L., Avolio B. J., Avolio B. J., Walumbwa F. O. (2005). *Authentic Leadership Theory And Practice: Origins, Effects, and Development*.

[12] Avolio B. J., Gardner W. L. (2005). Authentic leadership development: getting to the root of positive forms of leadership. *The Leadership Quarterly*.

[13] Luthans F., Avolio B. J., Cameron K. S., Dutton J. E., Quinn R. E. (2003). Authentic leadership development. *Positive Organizational Scholarship*.

[14] Walumbwa F. O., Wang P., Wang H., Schaubroeck J., Avolio B. J. (2010). Retracted: Psychological processes linking authentic leadership to follower behaviors. *The Leadership Quarterly*.

[15] Luthans F., Youssef-Morgan C. M., Avolio B. J. (2015). *Psychological Capital and Beyond*.

[16] Bazeley P., Jackson P. (2013). *Qualitative Data Analysis with Nvivo*.

[17] Luthans F., Youssef C. M., Avolio B. J. (2007). *Psychological Capital*.

[18] May D. R., Chan A. Y. L., Hodges T. D., Avolio B. J. (2003). Developing the moral component of authentic leadership. *Organizational Dynamics*.

[19] Stajkovic A. D., Luthans F. (1998). Social cognitive theory and self-efficacy: going beyond traditional motivational and behavioral approaches. *Organizational Dynamics*.

[20] George W. (2003). *Authentic Leadership: Rediscovering The Secrets to Creating Lasting Value*.

[21] Gardner W. L., Avolio B. J., Luthans F., May D. R., Walumbwa F. (2005). Can you see the real me? A self-based model of authentic leader and follower development. *The Leadership Quarterly*.

[22] Stevens R. (1983). *Erik Erikson: An Introduction*.

[23] Erikson E. H. (1985). *The Life Cycle Completed: A Review*.

[24] Hoare C. H. (2000). *Morality, Ethics, Spirituality, and Prejudice in The Writings of Erik H. Erikson*.

[25] Archer S. L. (2002). Commentary on ‘Feminist perspectives on Erikson’s theory: their relevance for contemporary identity development research’. *Identity*.

[26] Korostelina K. V. (2007). *Social Identity and Conflict: Structures, Dynamics and Implications*.

[27] Taylor B. (2003). *Modern Social Imagineries*.

[28] Bujo B. (2003). *Foundations of an African Ethic: Beyond The Universal Claims of Western Morality*.

